# Adjuvant Pericardial Sac Restraining in Heart Failure Treatment. A Medical Hypothesis Illustrated by a Case Report

**DOI:** 10.5935/1678-9741.20160004

**Published:** 2016

**Authors:** Paulo Roberto Barbosa Evora, Minna Moreira D. Romano, Luis Gustavo Gali, André Schmidt, Alfredo José Rodrigues

**Affiliations:** 1 Departament of Anatomy at Ribeirão Preto Medical School of the University of São Paulo (FMRP-USP), Ribeirão Preto, SP, Brazil.; 2 Departament of Internal Medicine at Ribeirão Preto Medical School of the University of São Paulo (FMRP-USP), Ribeirão Preto, SP, Brazil.

**Keywords:** Heart Failure, Pericardium, Cardiovascular Surgical Procedures, Cardiopulmonary Bypass

## Abstract

Ventricular constraint therapy has been used to prevent and reverse the progression of heart failure in ischemic and nonischemic cardiomyopathies. We hypothesized that ventricular restraint should be tried by closing the pericardium that was previously opened following left ventricle topographical projection. The surgical technique presentation is illustrated by a remarkable 13-year outcome of one patient with dilated cardiomyopathy treated surgically by mitral prosthesis, Cox/Maze III surgery to treat atrial fibrillation, and associated to the ventricular constraint using the patient's own pericardium. The ventricular pericardial restraint role is unclear, since the patient had multiple corrections that could be responsible for the good outcome; however it is viable deserving investigations.

**Table:** 

**Abbreviations, acronyms & symbols**	
AF	= Atrial fibrillation
CI	= Cardiac index
DC	= Dilated cardiomyopathy
EF	= Ejection fraction
HF	= Heart failure
LA	= Left atrium
LV	= Left ventricle
LVEDV	= Left ventricular end-diastolic volume
MLHF	= Minnesota Living with Heart Failure
MV	= Mitral valve
NYHA	= New York Heart Association
MVI	= Mitral valve insufficiency

## INTRODUCTION

Dilated cardiomyopathy (DC) is one of the most serious cardiovascular diseases, leading to sustained and increased morbidity rates. It is a public health issue associated with poor outcomes in the adult population and has become the leading cause of death in adults^[1]^.

The incidence of heart failure (HF) in the United States has been increasing, with 825,000 new cases in 2013. Despite medical and surgical advances, 50% of patients diagnosed with HF die within five years^[2]^. This syndrome has many idiopathic causes as well as recognized etiologies, among which the most common is coronary artery disease. Other etiologies include tachycardia-induced cardiomyopathy, storage disorders, and metabolic disorders, viral and postpartum. As a consequence, the increased volume of the left ventricle (LV) causes wall stress and high energy expenditure, triggering a mechanism of positive feedback that leads to progressive cardiac remodeling, marked cardiomegaly, spherical LV deformation, and mitral valve insufficiency (MVI)^[2]^.

Although heart transplantation is still the gold standard of treatment for DC, most patients, such as those who are older adult patients and those with comorbidities or socioeconomic limitations, are excluded. In addition to neurohormonal blockade (developed in the latter years of the 20^th^ century), selected patients with advanced HF have several alternatives treatments, such as cardiac resynchronization therapy or left ventricular assist devices, as well as surgical procedures, including mitral valve (MV) surgery (valve replacement or repair) and partial left ventriculectomy. These treatments should be considered as alternatives or bridge therapies to orthotropic heart transplantation.

Ventricular constraint therapy has been used to prevent or reverse the progression of HF in ischemic and non-ischemic cardiomyopathies. Two devices have been used clinically: a polyester multifilament mesh (CorCap Cardiac Support Device, Acorn, St. Paul, MN, USA) and a nitinol mesh for ventricular wrapping (HeartNet device, Paracor Medical, Sunnyvale, CA, USA)^[3]^. We hypothesized that ventricular restraint should be tried by closing the pericardium that was previously opened following LV topographical projection.

## CASE ILLUSTRATION

A 54-year-old male patient was attended for the first time in 2002 for a history of progressive dyspnea that had progressed to resting dyspnea (NYHA class IV). Physical examination revealed cardiac atrial fibrillation (AF), heart rate 160 bpm, jugular stasis, and a palpable liver situated at 3-4 cm from the right costal margin. He had a history of rheumatic heart disease, alcoholism, and smoking. An initial echocardiogram revealed the presence of mild deficiencies of the mitral and tricuspid valves associated with dilation of the heart chamber and an ejection fraction (EF) of 20%.

During 18 months of nutritional and pharmacologically optimized treatment (consisting of the neurohormonal blockade, diuretics, digitalis and anticoagulant), the patient remained in the advanced stage of HF. This clinical situation was consistently associated with echocardiographic findings that were incompatible with the severity of his clinical status. Based on these indicators, we diagnosed the patient with dilated cardiomyopathy associated with severe HF, possibly indicating heart transplantation. The personal circumstances and difficulties inherent in transplant surgery, MV replacement, and treatment of AF were discussed. On that occasion, it was proposed that external ventricular constraint related to these procedures (using the Acorn CorCap device) could confer additional benefits. After extensive discussion with the heart team and the patient, it was decided to test the feasibility of external ventricular restriction using the patient's own pericardium. After obtaining the consent of the patient to this trial, we proceeded with the surgical treatment.

The surgical findings included the following: 1) atrial fibrillation (AF) rhythm; 2) great cardiomegaly at the expense mainly of the left atrium (LA) and LV; 3) cardiac index (CI) = 1.3; 4) mitral valve (MV) calcified and fibrotic at the edges with some shortening or lengthening chordal; and 5) a great left atrial appendage without thrombi. Under cardiopulmonary bypass and myocardial protection with blood cardioplegia, surgery was performed in four steps:

1. Pericardiotomy following the topography of the heart - Preserving pleural integrity, we proceeded to perform a vertical pericardiotomy down to the level of the atrioventricular groove, diverting the incision obliquely toward the apex of the LV, as shown in Figure 1A (projection of the incision) and Figure 1B (pericardiotomy).
  **Fig. 1:**
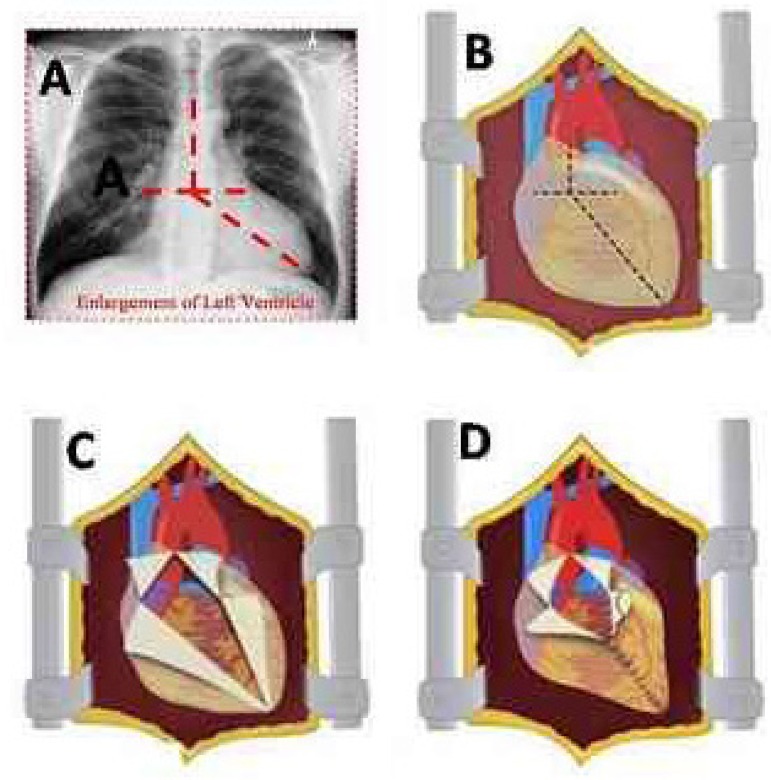
Surgical technique schematic presentation. A and B - Projection of the pericardial incisions; C - Pericardiotomy; D - Pericardium restraint suture.
2. MV replacement - We used a biological MV prosthesis consisting of bovine pericardium (Braile-M29) and preserved the valve apparatus by performing a resection of the anterior leaflet in a lunate shape, between the edge where the chordae were inserted and the anterior annulus. We implanted the prosthesis using 13 wires of Mersilene 2-0-anchored Teflon; the anterior leaflet points were passed at the free edge of the leaflet and the ring remained.
3. Cox/Maze III surgery for the treatment of AF - The third step comprised disconnecting and suturing the pulmonary veins; resecting and suturing the left atrial appendages, and making atrial incisions and sutures. After aortic clamp release, ventricular fibrillation reverted to sinus rhythm by way of internal defibrillation. The patient was discontinued from cardiopulmonary bypass with CI = 2.4, sinus rhythm, with 10 mcg/kg/min of dobutamine, withdrawal of cannulas, wire pacemaker in right atrium and right ventricle, and hemostasis.
4. Restraint of the ventricles- With the aid of the pericardium flaps, adjusted snugly by the surgeon at the end diastole, the surgeon narrowed the suture from the LV apex to the atrioventricular groove, referring to hemodynamic monitoring and completing the superior longitudinal pericardium incision without atrial constriction (Figure 1C and 1D). After this maneuver, the ventricles were constrained in a position where there was no hypotension or CI decrease. A suction drain was inserted into the pericardial sac.

**Fig. 2:**
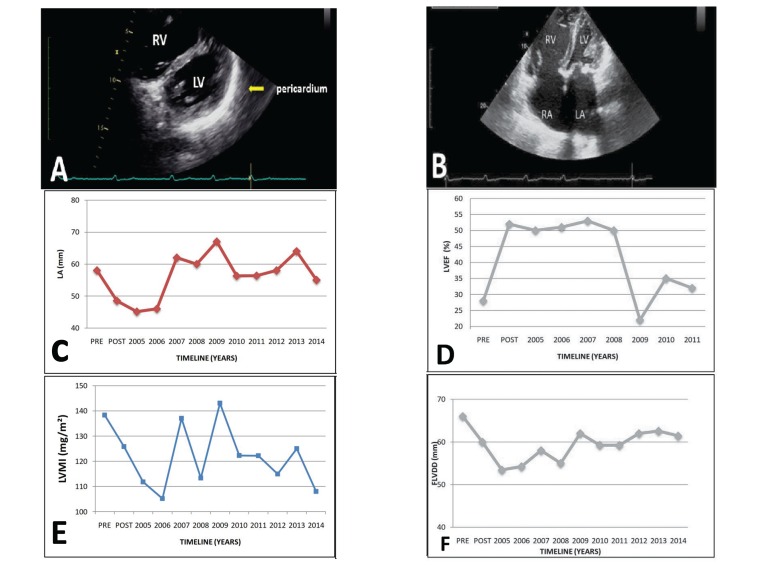
A - Twelve-years postoperative apical echocardiographic view showing a slightly decreased left ventricle volume (LV) while there is atrium and right ventricle (RV) dilation; B - Apical echocardiographic view of postoperative shows dilation of the right and left chambers; C - LA diameter timeline evolution; D - LVDD=left ventricle diastolic diameter timeline evolution; E - LVMI= left ventricular mass index timeline evolution, and; F - EF=ejection fraction timeline evolution

During the first two years after surgery, the patient's clinical outcome was associated with highly positive echocardiographic data that were consistently observed until the recurrence of AF. Even then, the patient's clinical course remained well-controlled (NYHA class II). In the eighth year after surgery, however, echocardiographic evidence began to show degeneration of the mitral prosthesis. The patient's last hospitalization resulted from severe gastrointestinal bleeding caused by the gastroduodenal artery and required blood transfusions. During hospitalization, the patient developed severe HF, and echocardiography showed marked degeneration with stenosis of the mitral prosthesis. This prosthesis was replaced by another bovine pericardial valve that was implanted via a transatrial approach, keeping the restrictive pericardium sac. The surgery and the early and four-month postoperative outcomes were uneventful. The echocardiogram timeline data is presented in Figure 2.

## DISCUSSION

The first part of this discussion briefly mentions the two well-known surgical approaches to treat HF that surely had capitol importance for the remarkable patient outcome: 1) The mitral prosthesis preserving the valve apparatus, and 2) The Cox/Maze III surgery to treat atrial fibrillation.

In 1984, a group at Harvard University postulated that the correction of MVI would increase systolic volume regardless of left ventricular EF. This concept became the impetus for using MV surgery as an adjunct HF treatment. This group's hypothesis was based on possible systolic volume increase, LV volume overload and end-diastolic pressure decrease^[4]^. Bolling et al.^[5]^, from the University of Michigan, proposed a technique for mitral annulus reduction using undersized rings in the repair of MVI. Their aim was to obtain the additive effect of reshaping the LV to facilitate the return of ventricular ellipsoidal conformation. In other studies, optimal functional improvement after partial ventriculectomy (the Batista operation) has been considered when mitral regurgitation is corrected simultaneously, thereby reinforcing this concept^[6,7]^.

The Cox/Maze III surgical procedure remains the treatment with the highest cure rates (over 90%), but the challenging technical nature of the traditional cut-and-sew technique has limited its mainstream uptake^[8]^.

Because of the enduring belief in its salutary effects on HF patients, strategies for achieving surgical ventricular restoration using fewer invasive methods continue to be pursued. A number of devices designed to restore LV geometry and decrease wall stress have been tested in both ischemic and nonischemic HF patients. Of all the devices developed to date, the most tested has been the CorCap Cardiac Support Device. The CorCap device consists of a polyester mesh that is placed circumferentially around the heart, from the apex to the atrioventricular groove. It provides diastolic resistance to filling by being adjusted snugly by the surgeon at the end diastole. In addition, it provides circumferential support, decreases LV wall stress, and resists progressive chamber dilation without any systolic assistance^[3]^. After impressive results in sheep models of ischemic cardiomyopathy, in which CorCap reduced left ventricular end-diastolic volume (LVEDV) by 39% and increased EF by 90%, phase 2 studies confirmed its safety and feasibility in human subjects. Subsequently, results from 300 HF patients in the Acorn Pivotal Trial were published, comparing CorCap with mitral surgery *versus* mitral surgery alone, and CorCap plus medical therapy *versus* medical therapy alone. In this trial, 148 patients were treated with CorCap. However, despite needing fewer subsequent procedures, having improved NYHA class and Minnesota Living with Heart Failure (MLHF) score and favorable echocardiographic reverse remodeling, patients did not demonstrate improvement in survival at 1, 3, or 5 years^[9]^.

Mortality rates remain significant in patients waiting for heart transplantation, perhaps because treatment alternatives for HF are still part of an open research field^[1,2]^. In planning the surgery, the above discussion took into account the hypothesized benefits of using an *in situ* patient pericardium to constrain the LV. Although the degree to which the *in situ* ventricular and pericardial restraint contributed to the patient's good outcome is unclear, since the patient had multiple corrections that could be responsible for the good result, however its presentation as a viable technique is pertinent to further investigations.

**Table:** 

**Authors' roles & responsibilities**	
PRBE	Conception and design study; realization of operations and/or trials; analysis and/or data interpretation; statistical analysis; manuscript writing or critical review of its content; final manuscript approval
MMDR	Realization of operations and/or trials; analysis and/or data interpretation; manuscript writing or critical review of its content; final manuscript approval
LGG	Realization of operations and/or trials; analysis and/or data interpretation; manuscript redaction or critical review of its content; final manuscript approval
AS	Analysis and/or data interpretation; manuscript redaction or critical review of its content; final manuscript approval
AJR	Realization of operations and/or trials; analysis and/or data interpretation; manuscript redaction or critical review of its content; final manuscript approval
